# Comparative physicochemical, hormonal, transcriptomic and proteomic analyses provide new insights into the formation mechanism of two chemotypes of *Pogostemon cablin*

**DOI:** 10.1371/journal.pone.0290402

**Published:** 2023-09-22

**Authors:** Hongyi Zhang, Xiaohua Ou, Wenyi Chen, Qing Zeng, Yaling Yan, Mengling He, Hanjing Yan

**Affiliations:** 1 College of Traditional Chinese Medicine, Guangdong Pharmaceutical University, Guangzhou, China; 2 Key Laboratory of State Administration of Traditional Chinese Medicine for Production & Development of Cantonese Medicinal Materials, Guangzhou, China; 3 Guangdong Provincial Research Center on Good Agricultural Practice & Comprehensive Agricultural Development Engineering Technology of Cantonese Medicinal Materials, Guangzhou, China; CSIR-Institute of Himalayan Bioresource Technology: Institute of Himalayan Bioresource Technology CSIR, INDIA

## Abstract

Patchouli (*Pogostemon cablin*) is an aromatic plant, and its oil has diverse applications in medicine, food, and cosmetics. Patchouli alcohol is the principal bioactive constituent of its volatile oil. In China, patchouli is typically categorized into two types: patchoulol-type (PA-type) and pogostone-type (PO-type). The study evaluated physiological and biochemical indicators, phytohormone metabolites and conducted transcriptome and proteome analyses on both two chemotypes. The PA-type exhibited higher levels of chlorophyll a, b, and carotenoids than the PO-type. In total, 35 phytohormone metabolites representing cytokinin, abscisic acid, gibberellin, jasmonic acid, and their derivatives were identified using UPLC-MS/MS, 10 of which displayed significant differences, mainly belong to cytokinins and jasmonates. Transcriptome analysis identified 4,799 differentially expressed genes (DEGs), while proteome analysis identified 150 differentially expressed proteins (DEPs). Regarding the transcriptome results, the DEGs of the PO-type showed significant downregulation in the pathways of photosynthesis, photosynthesis-antenna protein, porphyrin and chlorophyll metabolism, carotenoid biosynthesis, sesquiterpene and triterpenoid biosynthesis, and starch and sucrose metabolism, but upregulation in the pathway of zeatin synthesis. A combination of transcriptome and proteome analyses revealed that the DEGs and DEPs of lipoxygenase (LOX2), β-glucosidase, and patchouli synthase (PTS) were collectively downregulated, while the DEGs and DEPs of Zeatin O-xylosyltransferase (ZOX1) and α-amylase (AMY) were jointly upregulated in the PO-type compared to the PA-type. Differential levels of phytohormones, variations in photosynthetic efficiency, and differential expression of genes in the sesquiterpene synthesis pathway may account for the morphological and major active component differences between the two chemotypes of patchouli. The findings of this study offer novel perspectives on the underlying mechanisms contributing to the formation of the two patchouli chemotypes.

## Introduction

*Pogostemon cablin* (Blanco) Benth., commonly known as patchouli, belongs to the Lamiaceae family. Its oil is widely used in perfumes, cosmetics, and medicines as a fragrance [1, 2]. In China, patchouli is also utilized in traditional Chinese medicine and is primarily cultivated in Guangdong and Hainan Provinces. The oil of patchouli contains numerous essential compounds, including patchouli alcohol (PA), β-patchoulene, caryophyllene, α-guaiene, seychellene, β-guaiene, δ-guaiene, spathulenol, and pogostone [3, 4]. PA, being a sesquiterpene alcohol, is considered the most significant ingredient in patchouli oil [5]. Based on the ratio of PA and pogostone in the chemical composition, *P*. *cablin* can be classified into two chemotypes: patchoulol-type (PA-type) and pogostone-type (PO-type) [6]. In PA-type patchouli, the content of PA is much higher than pogostone content, while in PO-type patchouli, the content of PA is equal to or less than that of pogostone in the stems and leaves. These two chemotypes differ not only in their chemical composition and ratios [7] but also in their morphological characteristics [8, 9]. Typically, PA-type patchouli grows taller, has more branches, and exhibits better stress resistance. However, the molecular regulatory mechanisms and metabolite accumulation processes involved in chemotype formation are not yet fully understood. Therefore, a comparative analysis of the physiological and biochemical properties, phytohormone contents, transcriptome and proteome of these two chemotypes is of immense importance to gain insight into the formation mechanisms of the chemotypes.

## Materials and methods

### Plant treatment and sampling

The leaves and stems of PO-type and PA-type patchouli were used as test materials. The seedling samples were transplanted into the same size plastic pots filled with nutrient soil. Approximately 6 months later, the leaves and stems of the two chemotypes were collected and stored at -80°C for transcriptome and proteome analyses and data verification. Each of the two chemotypes were set up with three biological replicates.

### Estimation of physiological and biochemical parameters

Chlorophylls a, b and carotenoid were extracted according to Arnoff [10] with some modifications. 0.5 g of the samples were homogenized in 10 ml 80% acetone solution and centrifuged at 10,000 × g for 5 min. The quantification of carotenoids, chlorophyll a, and chlorophyll b was carried out by measuring the absorbance at 663 nm, 645 nm and 470 nm wavelengths, respectively, using a spectrophotometer. The concentrations of chlorophylls and carotenoids were calculated according to the equations provided by Lichtenthaler [11] and Porra [12] in mg/g fresh weight.

0.5 g of the samples were added to 4.5 mL solution consisting of 0.1 mol /L phos-hate buffer (pH 7.33) and ground to a powder in liquid nitrogen. After centrifugation at 3500 rpm for 10 minutes at 4°C, the supernatant was collected using commercial test kits (Nanjing Jiancheng Bioengineering Institute, Nanjing, China) to determine the contents of soluble protein, proline, and malondialdehyde (MDA), as well as the activity of peroxidase (POD) and superoxide dismutase (SOD) [13].

0.5 g of the samples were homogenized in 5 mL of distilled water and heated in a boiling water bath for 10 minutes. The mixture was then cooled to room temperature and centrifuged at 4,000 rpm for 10 minutes. The resulting supernatant was diluted 10 times with distilled water and thoroughly mixed by shaking to determine the soluble sugar content according to the instructions provided in the kit (Nanjing Jiancheng Bioengineering Institute, Nanjing, China).

The activity levels of SOD and POD were determined using the water-soluble tetrazolium salt-1 (WST-1) assay and colorimetry, respectively. The contents of MDA and soluble sugar were determined using the thiobarbituric acid assay and anthrone colorimetry, while the contents of soluble protein and proline were determined using Coomassie brilliant blue G-250 staining and ninhydrin colorimetry, respectively. All experiments were conducted three times with triplicates in each experiment.

### Phytohormone metabolites profiling

Sample preparation: The samples were ground into powder using a grinder (30 Hz, 1 min). 50 mg of the pulverized sample was accurately weighed and mixed with 10 μL of internal standard mixed solution (concentration of 100 ng/mL) and 1 mL of extraction solvent (methanol/water/formic acid 15:4:1, v/v/v). The mixture was vortexed for 10 min, followed by centrifugation at 12000 r/min for 5 min at 4°C. The supernatant was transferred to a new centrifuge tube for concentration. After that, 100 μL of 80% methanol/water solution was used for re-dissolution. The solution was filtered through a 0.22 μm membrane and placed in an injection bottle for UPLC-MS/MS analysis. The prepared sample solutions were analysed by an UPLC -MS/MS system (UPLC, SHIMADZU Nexera UHPLC LC-30A system; MS, Applied Biosystems QTRAP® 6500+) at VeryGenome Technology Co., Ltd. (Guangzhou, China).

The UPLC analytical conditions were as follows: UPLC column, Waters ACQUITY UPLC HSS T3 C18 (1.8 μm, 2.1 mm × 100 mm); solvent system, ultra-pure water (0.04% acetic acid): acetonitrile (0.04% acetic acid); gradient program, 0 min A/B at 95:5 (V/V), 1.0 min A/B at 95:5 (V/V), 8.0 min at 5:95 (V/ V), 9.0 min at 5:95 (V/V), 9.1 min at 95:5 (V/V), and 12.0 min at 95:5 (V/V); flow rate, 0.35 ml/min; temperature, 40 °C; and injection volume: 2 μl.

The mass spectrometry conditions mainly included: Electrospray Ionization (ESI) temperature at 550°C, the mass spectrometry voltage at 5500 V in positive mode, and -4500 V in negative mode, with Curtain Gas at 35 psi. Each ion pair was scanned and detected according to the optimized Declustering Potential (DP) and Collision Energy (CE) in Q-Trap 6500+.

The contents of phytohormones were calculated using the internal standard method and expressed as ng/g on a fresh weight basis.

### RNA isolation, RNA-sequencing and data analysis

Total RNA was extracted using a mirVana miRNA Isolation Kit (Ambion) following the manufacturer’s protocol. The integrity of RNA samples was evaluated using the Agilent Bioanalyzer 2100 System (Agilent Technologies, Santa Clara, CA, USA). The RNA integrity number values were above 7 for subsequent analysis. The libraries were constructed using the TruSeq Stranded mRNA LT Sample Prep Kit (Illumina, San Diego, CA, USA) according to the manufacturer’s instructions. Then, qualified cDNA libraries were constructed by an Illumina HiSeqTM 2500 instrument at Shanghai Eebiotech Company, and 125 bp or 150 bp paired reads were generated.

Raw data (raw reads) were processed using Trimmomatic v0.36 [14]. The clean reads were obtained by removing adaptor, ambiguous reads (‘N’) and low-quality sequences. Transcripts were assembled de novo by using Trinity v.2.4 [15] in the paired-end method. Transcriptomic data were submitted to the National Genomics Data Centre under accession number PRJNA884498.

To calculate transcription levels and differential transcription, FPKM values and DESeq v1.18.0 were used [16]. Genes with p value < 0.05 and |log2-fold change (logFC)| > 1 were deemed differentially expressed. GO and KEGG pathway enrichment analyses were performed to investigate the biological functions of DEGs using R based on the hypergeometric distribution [17].

### Protein extraction, digestion and TMT labelling

Pooled samples were used for proteomic analysis, the same as in RNA-Seq. Total protein was extracted from patchouli stem and leaf tissues, and the protein concentration was determined with BCA protein assay reagent (Beyotime Institute of Biotechnology, Shanghai, China).

The trypsin digestion protocol was performed as follows: first, the extracted proteins were reduced with 5 mM dithiothreitol for 30 min at 55°C. Next, each sample was alkylated with 10 mM iodoacetamide and incubated for 15 minutes at room temperature in the dark. Next, the proteins were digested with trypsin (enzyme/substrate ratio 1:50) overnight and then reconstituted with 100 mM triethylamine buffer (TEAB). Finally, the lyophilized samples were labelled using the 6 plex TMT reagent kit (Thermo Scientific, USA) following the manufacturer’s instructions.

### HPLC fractionation

Reversed-phase (RP) separations were performed on a 1,100 HPLC system (Agilent) with a 2.1 × 150 mm, 5 μm Agilent Zorbax Expense-C18 narrow-diameter column. The gradient elution system consisted of mobile phases A and ACN-H2O (2:98, V/V) and mobile phase B and ACN-H2O (90:10, v/v) with a flow rate of 300 μL/min and detection wavelengths of UV210 nm and 280 nm. The gradient program was set as follows: 0–8 min, 98% A; 8.00–8.01 min, 98–95% A; 8.01–48 min, 95–75% A; 60–60.01 min, 60–10% A; 60.01–70 min, 10% A; 70–70.01 min, 10–98% A; 70.01–75 min, 98% A. The samples were collected from 8 to 60 minutes, and elution buffer was collected every minute. Finally, the separated peptides were lyophilized for MS detection.

### LC–MS/MS analysis

The samples were tested by LC–MS/MS; the sample was loaded into an Acclaim Pepmap 100 100 μm × 2 cm precolumn (RP-C18, Thermo Fisher) at a flow rate of 300 nL/min and then separated by an Acclaim Pepmap RSLC 75 μm × 50 cm analytical column (RP-C18, Thermo Fisher).

The mass resolution of the primary MS was set to 70,000, and the automatic gain control (AGC) was set to 1e6 with a maximum injection time of 50 ms.

The mass spectrometry scan was set to the full scan charge-to-mass ratio m/z range of 300–1600, and the 10 peaks were scanned by MS/MS. MS/MS scanning was performed on the highest peak. All MS/MS spectra were collected using high-energy collision fragmentation in data-dependent positive ion mode, and the collision energy was set to 32.

The resolution of MS/MS was set to 17,500, and the AGC target was set to 2e5. The maximum injection time was 80 ms, and the dynamic exclusion time was set to 30.0 s.

Finally, Proteome Discover 2.4 (Thermo Fisher Company) data were used for protein analysis. TMT labelling, peptide fractionation and mass spectrometer detection were carried out by Shanghai Luming Biological Technology Co., Ltd. (Shanghai, China).

### Protein identification and bioinformatics analysis

Data analysis was performed by using Proteome Discover 2.4 (Thermo Fisher Corporation) software. After the original data were obtained by database retrieval, the confidently identified proteins were screened out according to the criteria of Score Sequest HT > 0 and unique peptide ≥ 1, and blank values were removed.

On the basis of the screened confidently identified proteins, the fold change in protein expression level between the samples and p value calculated by t-test were used to identify differentially expressed proteins (DEPs) (fold change ≥ 2 or < 0.5 and p-value < 0.05).

The STRING (https://string-db.org/) database was used to predict the functional correlation of DEPs.

### Integrated analysis of transcriptomic and proteomic data

An integrated analysis was performed to investigate the consistency between the proteomics and transcriptomics levels of the two chemotypes. Spearman’s correlation test was used to assess the correlation between transcriptome gene expression levels and the corresponding proteome proteins [18]. The results were divided into three categories: the expression trends of differentially expressed genes (DEGs) and DEPs were the same, the expression trends of DEGs and DEPs were opposite, and there was no difference in expression between DEGs and DEPs.

### Statistical analysis

The graphs in physiological and biochemical index testing were generated using GraphPad Prism (v. 9.3.1, GraphPad Software Inc., CA, USA), and statistical analysis was performed using a Student’s t-test for comparison between two sets of data. A p-value less than 0.05 (*P < 0.05) was considered statistically significant. All data shown were the mean values of three biological replicates (n  =  3).

## Results

### Analysis of physiological and biochemical indicators

**Fig 1:**
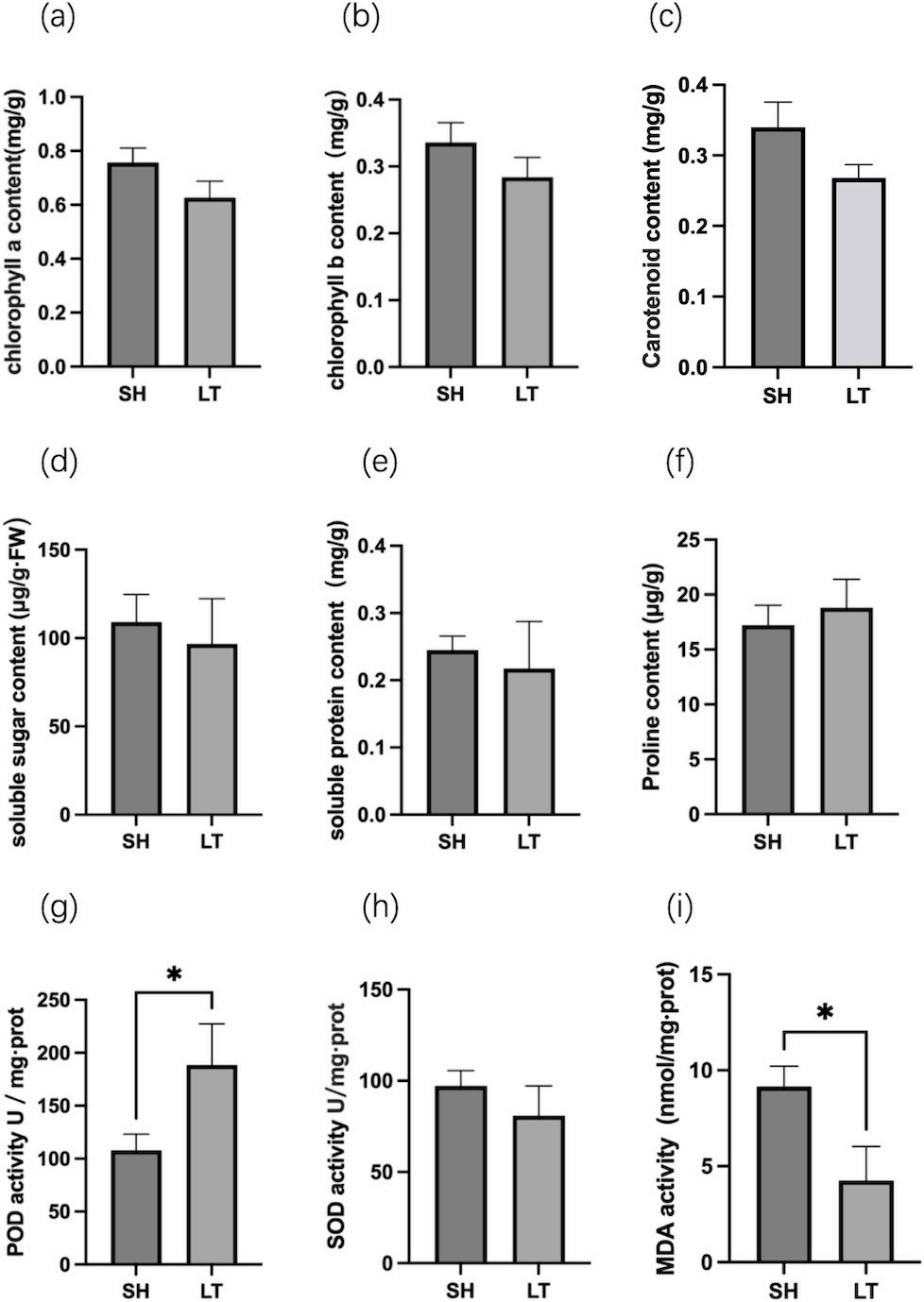
Analysis of physiological and biochemical indexes of two chemotypes of *P*. ***cablin*.** (a) Chlorophyll a content (b) Chlorophyll b content (c) Carotenoid content (d) Soluble sugar content (e) Soluble protein content (f) Proline content (g) POD activity (h) SOD activity (i) MDA activity Values shown were means ±SD from three biological replicates. Symbol * indicated that the significant differences between SH (the PA-type) and LT (the PO-type) at 0.05.

Based on these physiological and biochemical indicators, the two chemotypes of patchouli showed different levels of photosynthetic pigments and antioxidant enzyme activity (Fig 1). Compared with the PO-type, the PA-type had generally higher contents of chlorophyll a, b, carotenoids, and soluble sugars. On the other hand, the PO-type had higher content of soluble proteins than the PA-type. There were significant differences in POD and MDA activity between the two chemotypes. The POD activity of the PO-type was 1.82 times higher than that of the PA-type, indicating that the POD of the PO-type was more sensitive to external factors. In addition, the MDA activity of the PA-type was 2.29 times higher than that of the PO-type.

### Endogenous phytohormone metabolites profiling

We quantified cytokinins (CKs), gibberellic acids (GAs), abscisic acid (ABAs) and jasmonic acid (JAs) hormones, along with their major metabolites using UPLC-MS/MS. A total of 35 phytohormone metabolites were identified (S1 Table). We identified the final differential metabolites with log2foldchange ≥1 and P value ≤0.05. In total, there were 10 phytohormone metabolites identified (Table 1). Six differential phytohormone metabolites were significantly up-regulated in the PO-type compared to the PA-type, including 2-chloro-trans-zeatin (2CltZ), cis-zeatin-riboside (cZR), 2-methylthio-N6-isopentenyladenine riboside (2MeSiPR), GA3, jasmonoyl-L-isoleucine (JA-Ile), and meta-topolin-9-glucoside (mT9G). Additionally, four differential hormone metabolites were significantly downregulated including JA, 3-oxo-2-(2-(Z)-pentenyl) cyclopentane-1-butyric acid (OPC-4), dihydrojasmonic acid (H2JA), and N^6^-(Δ^2^-isopentenyl) adenine (IP). The results of the phytohormone metabolite analysis indicated that PO-type patchouli had higher levels of zeatin phytohormone metabolites, while PA-type patchouli had higher levels of JA phytohormone metabolites.

**Table 1:** Ten differential phytohormone metabolites of two chemotypes of *P. cablin*.

compound	LT-1	LT-2	LT-3	SH-1	SH-2	SH-3	log2 foldchange	up_down	pvalue
H2JA	0.40335	0.57449	0.42226	1.76466	1.50478	1.3648	-1.7268029	down	0.00453136
IP	0.50413	0.4751	0.48955	1.53136	1.3774	1.31893	-1.5252513	down	0.00416537
JA	134.758	132.1849	132.1308	395.7653	372.1345	367.7658	-1.5088106	down	0.00113706
OPC-4	522.7474	469.0876	497.3653	1909.5041	1657.2349	1750.4284	-1.8361201	down	0.00236425
2CltZ	0.40634	0.46357	0.44139	0.18901	0.19372	0.13927	1.32887607	up	0.0004042
2MeSiPR	0.67434	0.65517	0.70608	0.25116	0.35726	0.30529	1.15563876	up	0.00190729
cZR	0.26829	0.29909	0.30288	0.13906	0.13249	0.13623	1.09365547	up	0.00407121
GA3	35.14438	38.69633	36.3967	14.13767	13.24747	13.11894	1.44447475	up	0.00090134
JA-ILE	18.26739	18.22434	18.36934	5.14118	5.02275	4.88683	1.86594642	up	2.30E-07
mT9G	3.22248	3.41648	3.12006	1.50101	1.70753	1.66387	1.00210074	up	0.00019991

Note: The samples of LT-1, LT-2, LT-3 were the PO-type patchouli and the samples of SH-1, SH-2, SH-3 were the PA-type patchouli.

### De novo assembly and annotation of transcriptomes

A total of 35.73 Gb of clean data was obtained by transcriptome sequencing with at least 93.9% Q30 from each sample (Table 2). All clean data were combined into 54,630 unigenes with a mean length of 1,026.2 bp.

**Table 2:** Quality control analysis for RNA-seq data.

Sample	RawReads/M	RawBases/G	CleanReads/M	CleanBases/G	ValidBases/%	Q30/%	GC/%
TLT-1	46.25	6.94	45.38	6.37	91.78	94.24	46.66
TLT-2	46.26	6.94	45.33	6.33	91.28	94.01	46.39
TLT-3	42.13	6.32	41.36	5.80	91.79	94.14	46.43
TSH-1	43.14	6.47	41.46	5.23	80.86	96.06	47.27
TSH-2	46.02	6.90	45.08	6.26	90.61	93.90	46.85
TSH-3	41.83	6.27	41.00	5.74	91.53	94.22	46.96

Note: The samples of TLT-1, TLT-2, TLT-3 were PO-type patchouli and the samples of TSH-1, TSH-2, TSH-3 were PA-type patchouli.

Common functional database annotations were performed on unigenes; 37,145 (67.99%) unigenes were annotated to the NR library, 27,250 (49.88%) unigenes were annotated to the SwissProt library, 13,015 (23.82%) unigenes were annotated to the Kyoto Encyclopedia of Genes and Genomes (KEGG) library, and 24,662 (45.14%) unigenes were annotated to the Gene Ontology (GO) library (Table 3).

**Table 3:** Functional database annotation results.

Anno_Database	Annotated_Number	300< = length<1000	length> = 1000
NR	37145(67.99%)	19237(35.21%)	17908(32.78%)
Swissprot	27250(49.88%)	12743(23.33%)	14507(26.56%)
KEGG	13015(23.82%)	6497(11.89%)	6518(11.93%)
KOG	21571(39.49%)	10433(19.10%)	11138(20.39%)
eggNOG	34035(62.30%)	16870(30.88%)	17165(31.42%)
GO	24662(45.14%)	11610(21.25%)	13052(23.89%)
Pfam	23439(42.90%)	9550(17.48%)	13889(25.42%)

**Fig 2:**
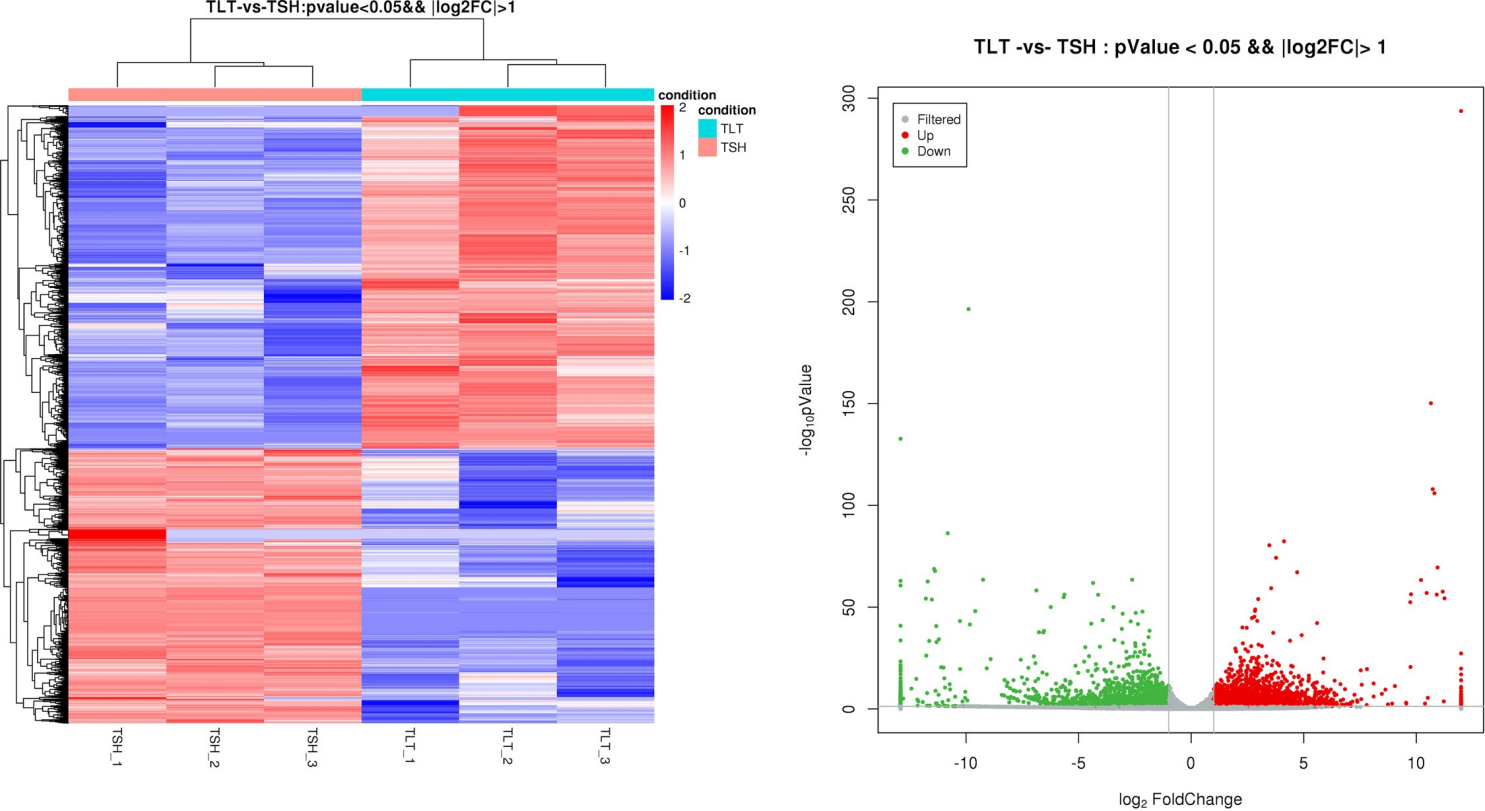
Heatmap and volcano plot of DEGs of two chemotypes. (a) DEG heatmap. (b) Volcano plot of DEGs of two chemotypes.

The DEGs of PA-type (TSH group) and PO-type (TLT group) were compared and analysed according to the conditions of p value < 0.05 and fold change > 2, and a heatmap and volcano plot were drawn to understand the DEGs of the two chemotypes (Fig 2). There were a total of 4,799 DEGs, including 2,665 upregulated DEGs and 2,134 downregulated DEGs.

### GO enrichment analysis of DEGs

**Fig 3:**
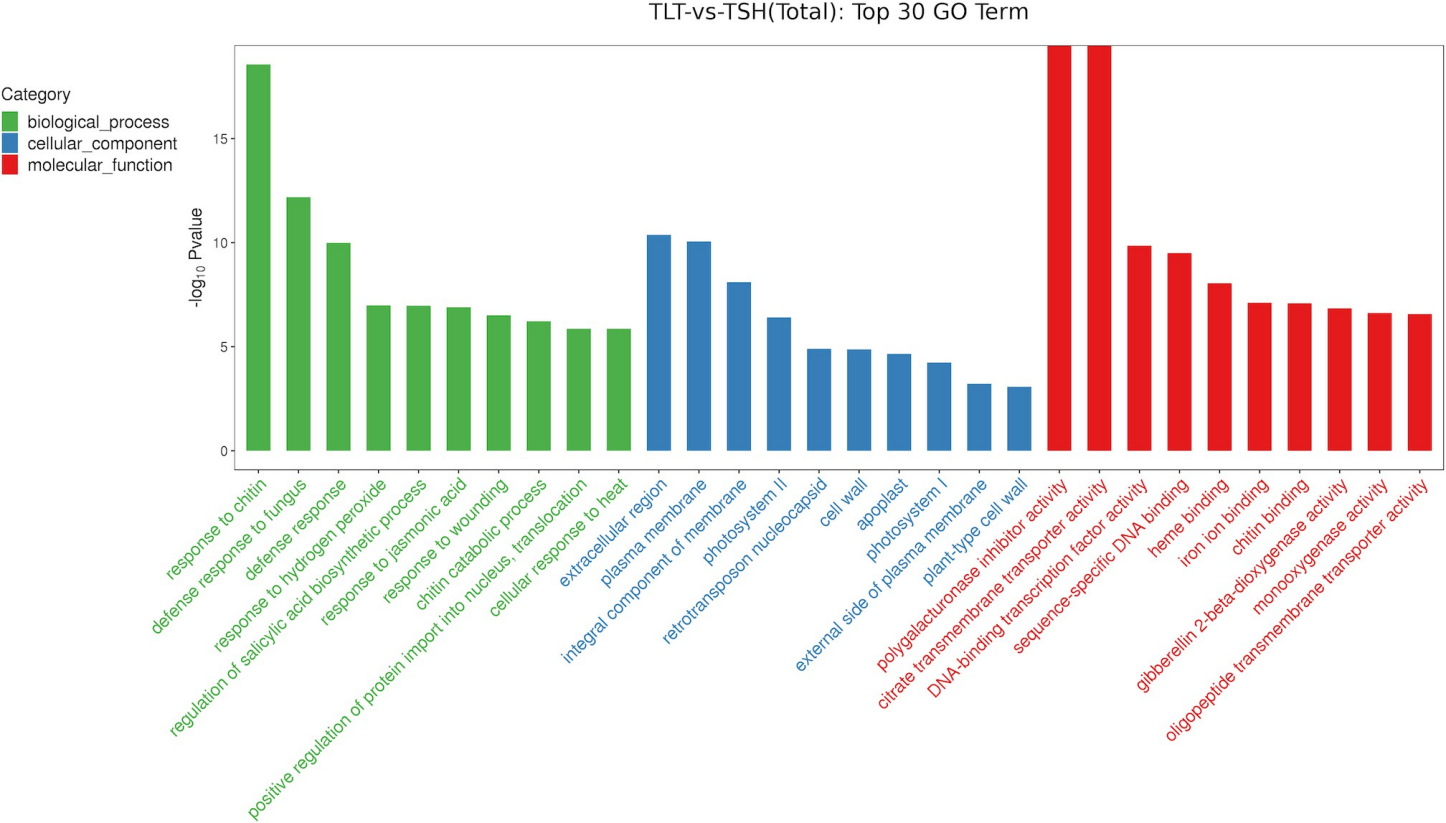
GO functional classification of DEGs.

A total of 2,608 DEGs were annotated in the GO database, including 1,580 upregulated and 1,028 downregulated DEGs in the TLT group compared to the TSH group. The DEGs involved in biological processes were significantly enriched in response to chitin, defence response to fungus, defence response, and response to hydrogen peroxide. The DEGs involved in cellular components were significantly enriched in extracellular region, plasma membrane, integral component of membrane, and photosystem II. The DEGs involved in molecular functions were significantly enriched in polygalacturonase inhibitor activity, citrate transmembrane transporter activity, DNA-binding transcription factor activity and sequence-specific DNA binding (Fig 3).

### KEGG enrichment analysis of DEGs

**Fig 4:**
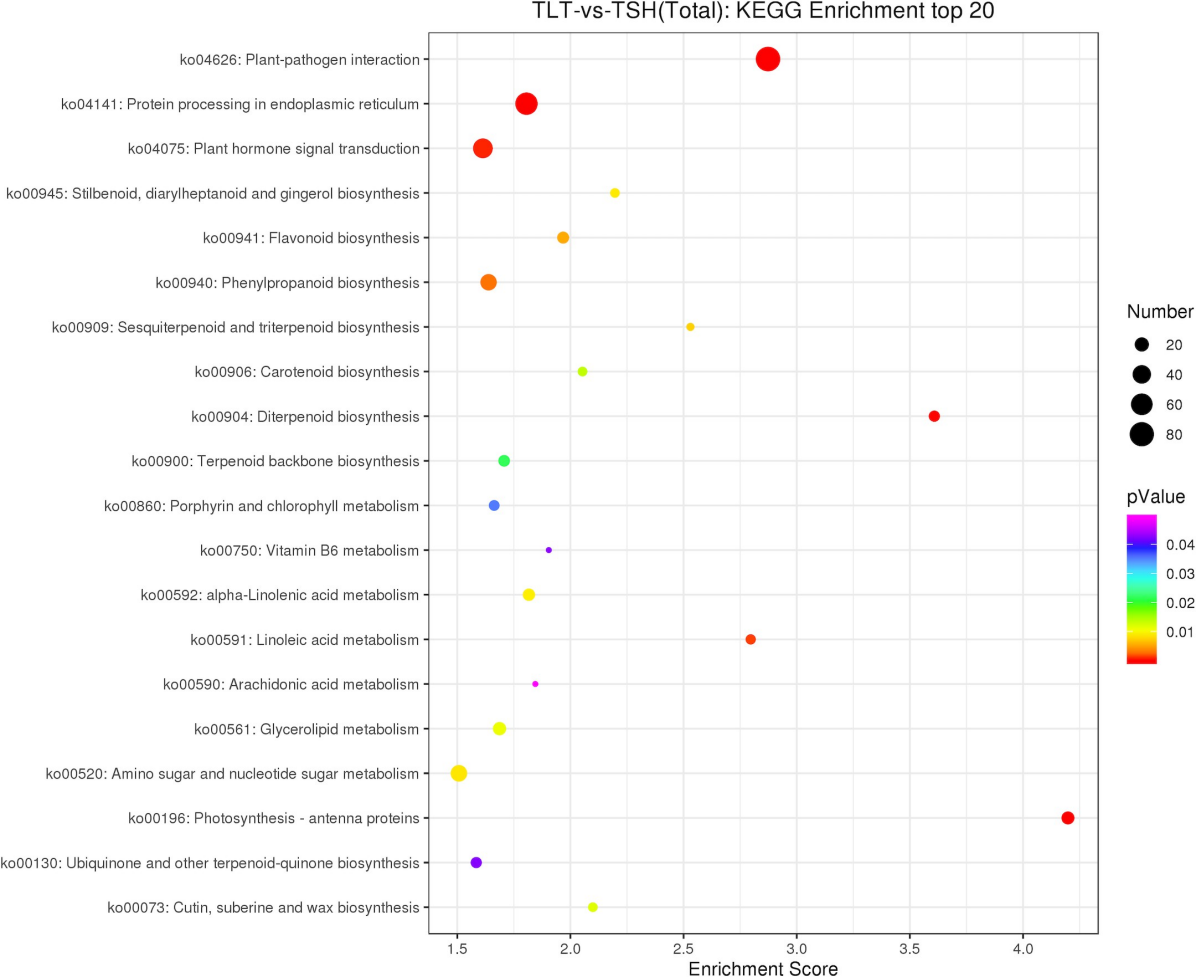
KEGG pathway analysis of DEGs. The vertical axis represents the pathway name, the horizontal axis represents the rich factor, the size of the dots represents the number of DEGs in the pathway, and the colour of the dots corresponds to different q-value ranges.

A total of 688 DEGs were annotated to the KEGG database and mapped onto 110 pathways. The enriched pathways included plant‒pathogen interaction (ko04626), photosynthesis-antenna proteins (ko00196), sesquiterpenoid and triterpenoid biosynthesis (ko00909), terpenoid backbone biosynthesis (ko00900), carotenoid biosynthesis (ko00906), diterpenoid biosynthesis (ko00904), plant hormone signal transduction (ko04075) and linoleic acid metabolism (ko00591) (Fig 4).

### Protein identification and DEP analysis

**Fig 5:**
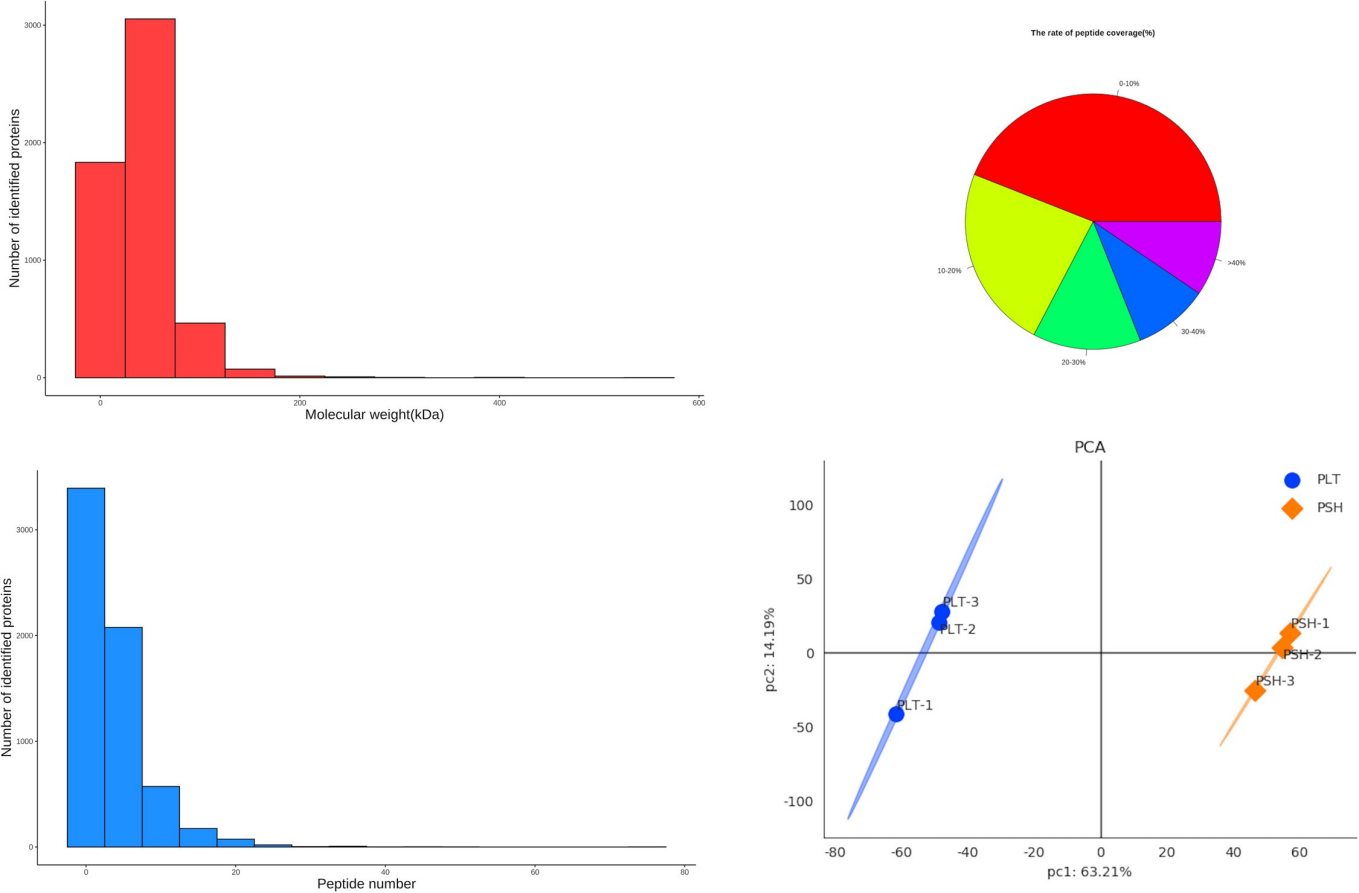
Protein identification and PCA analysis. (a) Molecular weight (b) Peptide number (c) Peptide sequence coverage (d) PCA analysis.

Proteome analysis of the two chemotypes was performed by TMT labelling technology. In total, 276,419 secondary spectra and 24,279 unique peptides were detected. After removing repetitive proteins, 5,451 proteins were identified based on TMT data. The statistical information of the identified proteins is shown in Fig 5, including molecular weight, peptide sequence length, distribution of peptide number, and protein quantification. Principal component analysis (PCA) was performed to evaluate the test samples. The results showed that the different groups of patchouli were well distinguished, and three repeated samples of each group were aggregated together (Fig 5D).

**Fig 6:**
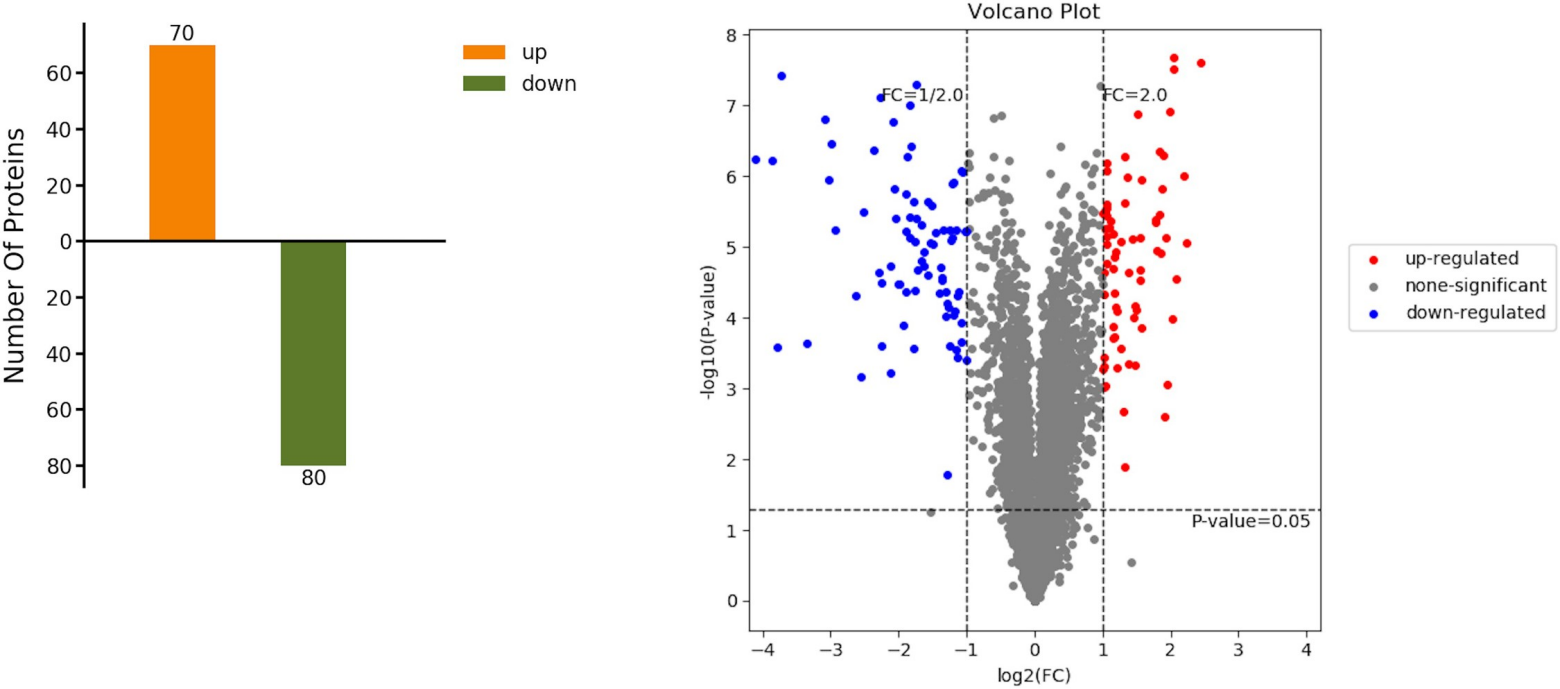
Statistic of DEPs. (a) Statistics of upregulated and downregulated DEGs (b)Volcano plot of DEPs. Each point in the differential expression volcano plot represents a protein; log2(FC): the logarithm of the fold difference in expression of a protein in two samples; −log10(p-Value): the negative logarithm of the statistical significance of the change in protein expression. The blue dots in the figure represent downregulated DEPs, the red dots represent upregulated DEPs, and the grey dots represent non-DEPs.

Comparing PO-type patchouli with PA-type patchouli, we discovered a total of 150 DEPs upon the upregulated and downregulated thresholds, with a fold change ≥2 or <0.5 and p < 0.05, with 70 upregulated and 80 downregulated (Fig 6).

**Fig 7:**
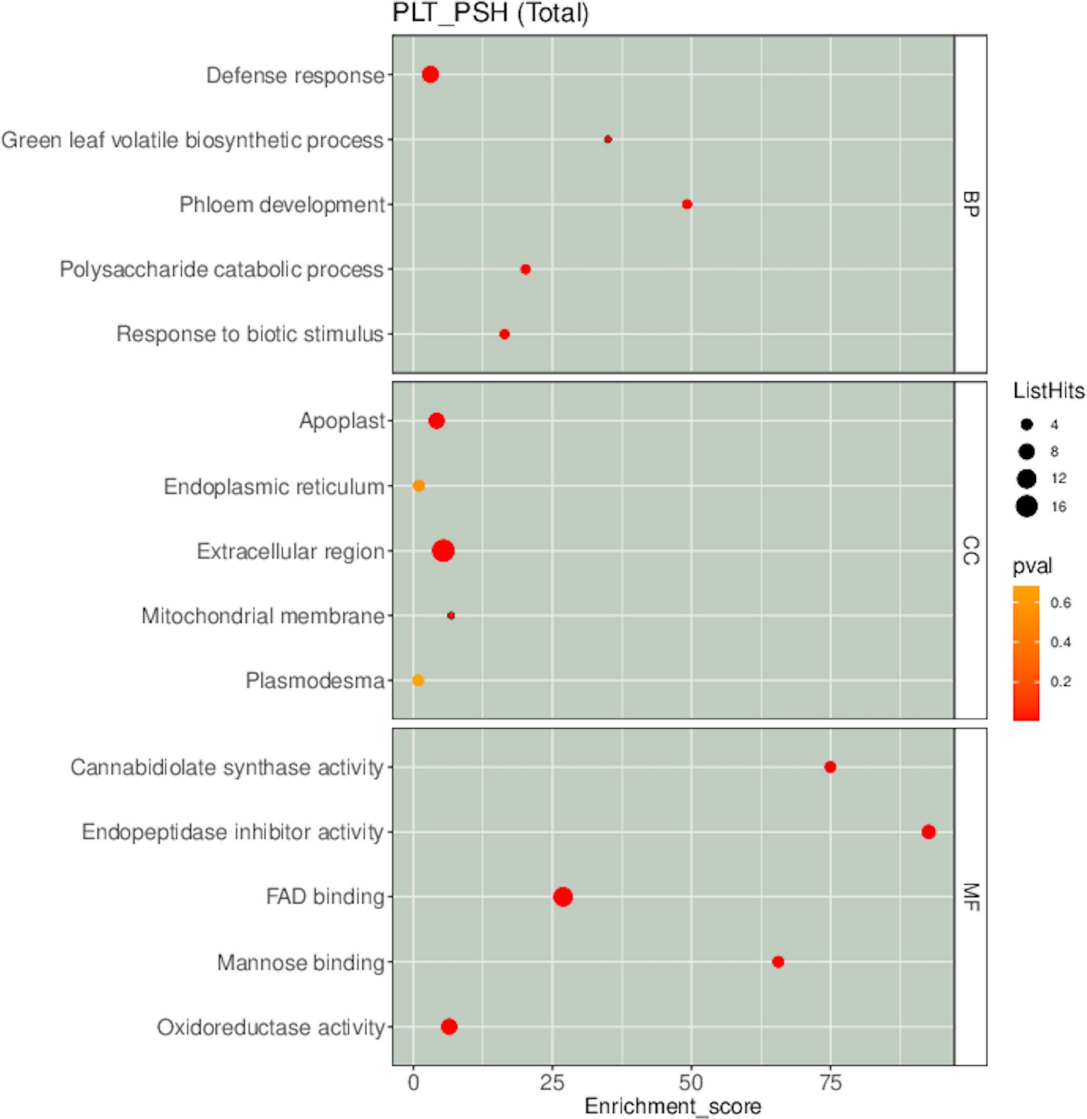
Go annotation of DEPs.

GO annotation showed that the DEPs were mainly enriched in the defense response and green leaf volatile biosynthetic process in biological processes, extracellular region, apoplast and mitochondrial membrane in cellular components and endopeptidase inhibitor activity, cannabidiolate synthase activity and FAD binding in molecular functions (Fig 7).

**Fig 8:**
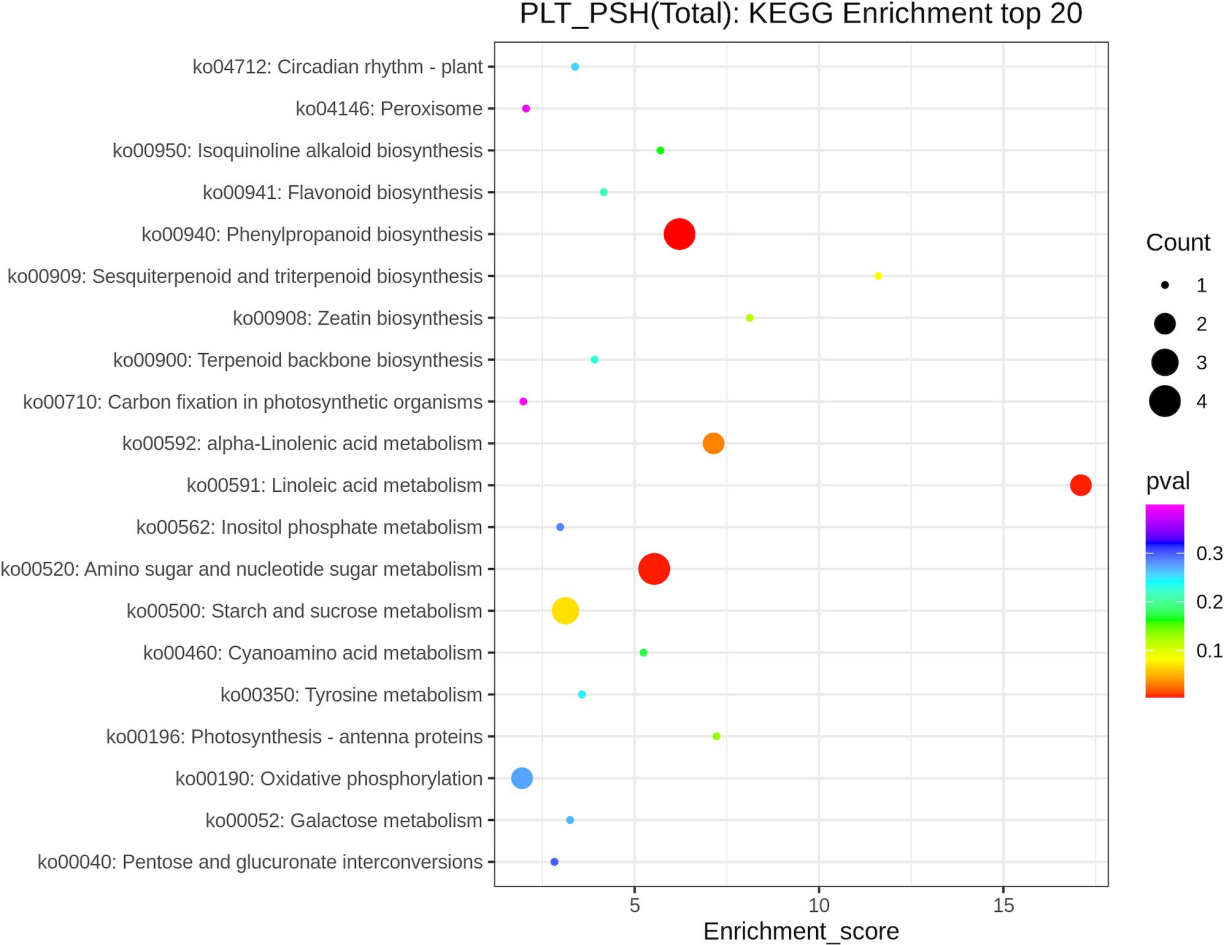
KEGG enrichment analysis of DEPs. The vertical axis represents the pathway name, the horizontal axis represents the rich factor, the size of the dots represents the number of DEPs in the pathway, and the colour of the dots corresponds to different p-value ranges.

A total of 25 DEPs belonging to 23 pathways were annotated into the KEGG database. The KEGG enrichment pathways of the two chemotypes were enriched in phenylpropanoid biosynthesis (ko00940), sesquiterpenoid and triterpenoid biosynthesis (ko00909), zeatin biosynthesis (ko00908), terpenoid backbone biosynthesis (ko00900), carbon fixation in photosynthetic organisms (ko00710), linoleic acid metabolism (ko00591), amino sugar and nucleotide sugar metabolism (ko00520), and photosynthesis-antenna proteins (ko00196) (Fig 8).

**Fig 9:**
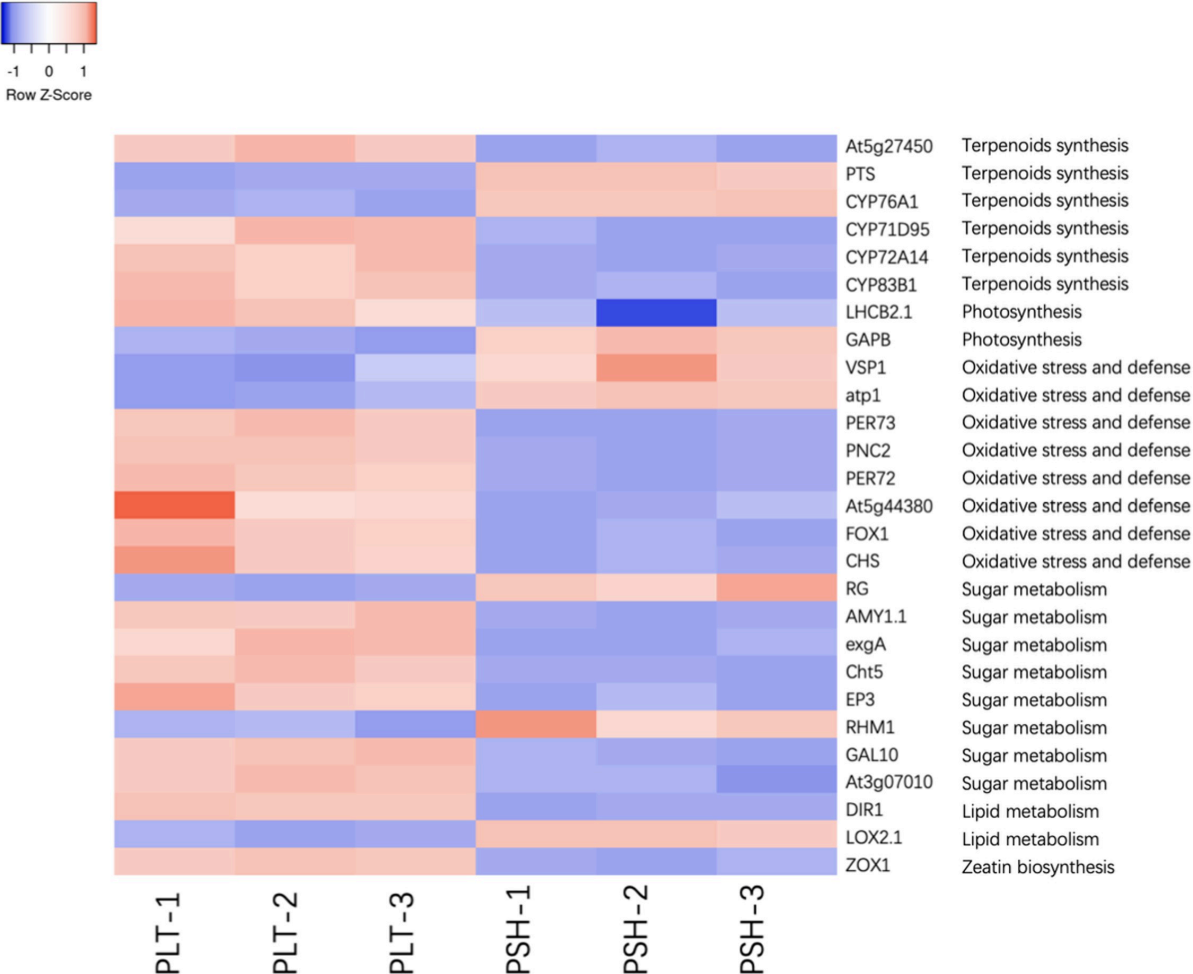
Heatmap of important DEPs of two chemotypes.

According to the results of the GO, KEGG and STRING databases, 27 important DEPs were further detected and were mainly involved in terpenoid synthesis, photosynthesis, oxidative stress and defense, sugar metabolism, lipid metabolism and zeatin biosynthesis (Fig 9).

**Fig 10:**
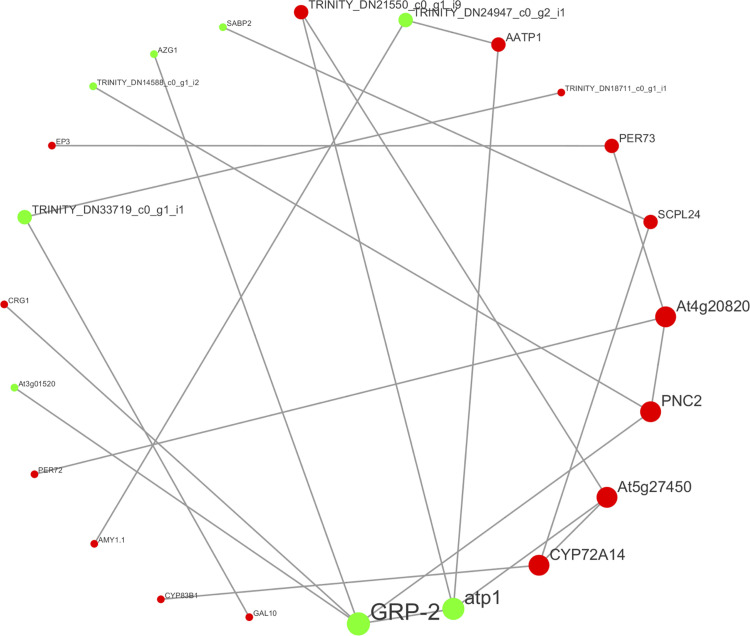
Protein–protein interaction maps of DEPs. The red color represents the uregulated proteins, and the green color represents the downregulated proteins.

The functional correlation prediction analysis of DEPs was carried out through STRING (https://string-db.org/) database. The interaction network analysis of the top 25 DEPs in terms of connectivity was performed using Cytoscape 3.7.2 software. The results showed GRP-2 (Glycine-rich protein 2) and atp1 (ATP synthase subunit alpha), At4g20820 (Berberine bridge enzyme-like 18), PNC2 (Cationic peroxidase 2), CYP72A14 (Cytochrome P450 72A14), At5g27450 (Mevalonate kinase) node proteins had a large number of interacting proteins (Fig 8). Interactions were found between downregulated DEP (atp1) and 1 downregulated DEP (GRP-2) as well as 3 upregulated DEPs (AATP1, Trinity_DN21550_c0_g1_i9, At5g27450) (Fig 10). The results suggested that these DEPs could interact to promote the expression of related genes and the accumulation of key metabolites.

### Transcriptome and proteome integrated analysis

The integrated analysis of the transcriptome and proteome data of the two chemotypes revealed a positively correlated relationship between mRNA and protein expression with a Pearson’s correlation coefficient of 0.6149, of which 0.1002 was significantly positively correlated (S1 Fig).

**Fig 11:**
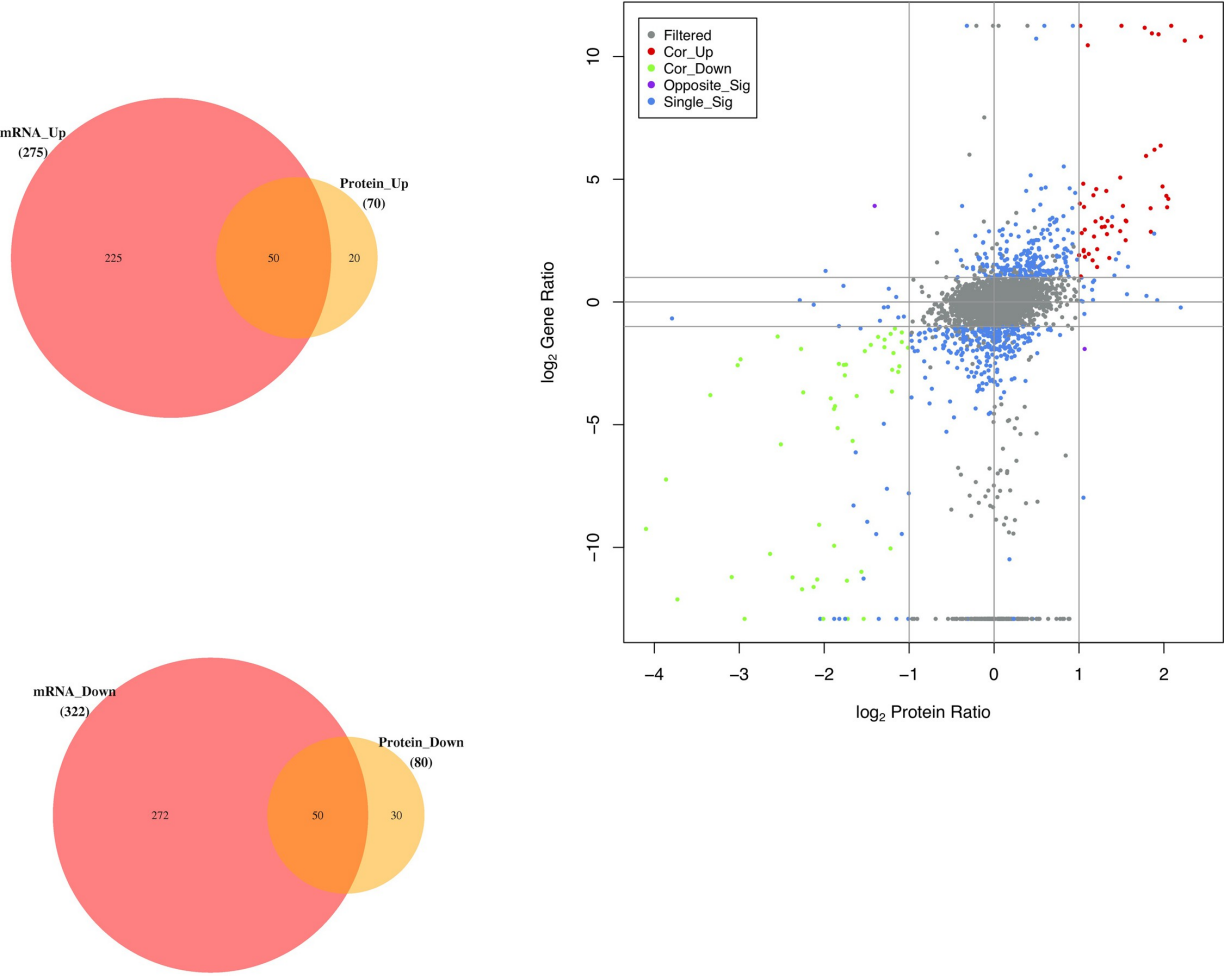
(a) Venn diagram of co-upregulated DEGs and DEPs. (b) Venn diagram of co-downregulated DEGs and DEPs. (c) Integrated analysis scatter plot of DEPs and DEGs. The horizontal and vertical coordinates represent the log2 value of the differential fold of protein and gene expression respectively; the red dots (Cor_Up) represent the DEPs/DEGs that were upregulated at the same time; the green dots (Cor_Down) represented the DEPs/DEGs that were downregulated at the same time; Purple dots (Opposite_Sig) represent DEPs/DEGs with opposite expression trends; blue dots (Single_Sig) represent DEPs/DEGs that are differentially expressed at only one level in protein or mRNA; grey dots (Filtered) represent non-DEPs/non-DEGs.

Fifty DEGs/DEPs showed upregulated expression, and 50 DEGs/DEPs showed downregulated expression at both the mRNA and protein levels. Two DEGs/DEPs showed opposite expression, and 543 DEGs/DEPs showed differential expression at only one level of protein or mRNA (Fig 11).

Meanwhile, to investigate the role of posttranscriptional regulation, GO and KEGG enrichment analyses were performed. The GO-enriched pathways included phenylpropanoid biosynthesis, starch and sucrose metabolism, zeatin O-beta-D-xylosyltransferase activity, and amino sugar and nucleotide sugar metabolism (S1 File).

There were 8 upregulated DEGs and DEPs significantly enriched in 8 KEGG pathways, including zeatin biosynthesis, circadian rhythm-plant, phenylpropanoid biosynthesis, amino sugars and nucleosides, and starch and sucrose metabolism (S2 File). In total, 7 downregulated DEGs and DEPs were significantly enriched in 10 KEGG pathways, including sesquiterpene and triterpenoid biosynthesis, linoleic acid metabolism, inositol phosphate metabolism, starch and sucrose metabolism, phenylpropanoid biosynthesis, and amino sugar and nucleotide sugar metabolism (S2 File).

Compared with PA-type, lipoxygenase (LOX2) DEGs/DEPs were downregulated in linoleic acid metabolism and alpha-linolenic acid metabolism of PO-type. Alpha-amylase (AMY) DEGs/DEPs were co-upregulated in starch and sucrose metabolism; however, beta-glucosidase and glucose-1-phosphate adenylyltransferase (glgC) were co-downregulated. Zeatin O-xylosyltransferase (ZOX1) DEGs/DEPs were upregulated in zeatin biosynthesis. In the terpenoid synthesis pathway, patchoulol synthase (PTS) DEGs/DEPs were downregulated.

## Discussion

To explore the molecular formation mechanism of the two chemotypes, we performed physiochemical, hormonal, transcriptomic and proteomic analyses. The PA-type exhibited higher levels of chlorophyll a, b, and carotenoids than the PO-type. In total, 35 phytohormone metabolites representing CKs, ABAs, GAs, JAs, and their derivatives were identified using UPLC-MS/MS, 10 of which displayed significant differences, mainly belong to CKs and JAs. A total of 4,799 DEGs were mainly involved in plant‒pathogen interactions, photosynthesis-antenna proteins, diterpenoid biosynthesis, plant hormone signal transduction, linoleic acid metabolism, and sesquiterpenoid and triterpenoid biosynthesis, while 150 DEPs were mainly involved in linoleic acid metabolism, phenylpropanoid biosynthesis, amino sugar and nucleotide sugar metabolism, alpha-linolenic acid metabolism, and sesquiterpenoid and triterpenoid biosynthesis.

The main component of patchouli oil is sesquiterpenoids, with PA being the predominant compound. In transcriptome KEGG enrichment analysis, the sesquiterpenoid and triterpenoid biosynthesis pathways and terpenoid skeleton biosynthesis pathway were significantly enriched. PTS is a sesquiterpene cyclase and a key enzyme in the synthesis of PA and can catalyse the conversion of farnesyl diphosphate (FPP) to PA plus at least 13 additional sesquiterpene products [19, 20]. PTS DEPs and DEGs were both downregulated in PO-type patchouli and upregulated in PA-type patchouli, which could lead to the lower content of sesquiterpenoids, such as patchouli, synthesized in PO-type patchouli than in PA-type patchouli. These results highlighted the crucial regulatory role of PTS in the synthesis of sesquiterpenoids in patchouli oil, with its gene and protein expression level showing a clear correlation with the chemical types of patchouli.

JA and its derivatives are plant hormones that act as signaling compounds in the regulation of cellular defense and development. Quantitative analysis of JA phytohormone metabolites indicated that the levels of JA, OPC-4, and H2JA were higher in the PA-type. These results suggested that the PA-type possibly exhibited a relatively higher content of JAs. LOXs are oxidoreductases found widely in plants and play a crucial role in JA biosynthesis. They utilize linoleic and linolenic acids as substrates to mediates the biosynthesis of JA [21, 22]. In *Arabidopsis*, AtLOX2 is responsible for JA biosynthesis [23]. Silencing of pepper CaLOX2 confirmed its involvement in JA biosynthesis [24]. External application of MeJA can induce the expression of MaLOX1 and MaLOX2, enhance the endogenous JA content, and improve the stress resistance of banana [25]. Furthermore, the transcriptomic and proteomic co-analysis revealed downregulation of LOX2 DEGs/DEPs in the linoleic acid metabolism and alpha-linolenic acid metabolism pathways specifically in the PO-type when compared to the PA-type. This result was consistented with the quantification of JA phytohormone metabolites in the two chemotypes, further highlighting the importance of LOXs in JA biosynthesis. Interestingly, external application of JA has been found to help increase the content of PA [1]. Additionally, the JA signaling pathway indicated upregulated expression of four jasmonate ZIM domain-containing protein (JAZ DEGs) in the PO-type. JAZ proteins have been identified as critical negative regulators in the JA signaling pathways of plants, and their involvement in JA-mediated secondary metabolism has been well-documented [26,27]. In *P*. *cablin*, PatJAZ6 is thought to act as a repressor in the biosynthesis of patchouli alcohol by JA signaling pathway [28]. Taken together, these findings suggested that JA and its derivatives likely played a more important role in the biosynthesis of PA and defense response in the PA-type than in the PO-type.

CKs, as important plant hormones, play a crucial role in cell division and plant growth [29] Among the various CKs, zeatin is recognized as a vital CK in higher plants due to its widespread occurrence and high activity [30, 31]. A total of 19 cytokinin metabolites were detected in patchouli through quantitative UPLC-MS/MS analysis, with significant differences observed for 5 cytokinin metabolites, including 2 zeatin metabolites (2CltZ and cZR). These two zeatin metabolites, along with 2MeSiPR and mT9G, were found to be more abundant in the PO-type, while only IP was more abundant in the PA-type. Meanwhile, upregulation of zeatin O-xylosyltransferase (ZOX1) DEGs/DEPs was observed during zeatin biosynthesis in the PO-type. The results from transcriptomics, proteomics, and CK metabolomics were consistent, indicating that zeatin was a common CK in patchouli and affected the growth and development of both chemotypes of patchouli.

Besides plant hormones, photosynthesis is essential for plant growth and yield [32]. Approximately 95% of the accumulation of plant biomass is due to photosynthesis [33]. It is driven by photosystem I (PSI) and photosystem II (PSII), which are two multisubunit complexes that are embedded in the thylakoid membrane of plants [34]. Light-harvesting complex I (LHCI) in the PSI antenna complex consists of four distinct subunits named LHCA1 to LHCBA4, while LHCII consists of six distinct subunits (LHCB 1 to LHCB 6) in the PSII antenna complexes [35, 36]. In PO-type patchouli, 4 DEGs encoding photosynthesis-antenna proteins, including LHCA2, LHCA3, and LHCA4 in PSI, and 12 DEGs encoding photosynthesis-antenna proteins, including LHCB1, LHCB3, and LHCB6 in PSII, were significantly downregulated. In addition, 6 DEGs encoding photosynthesis electron transporters (PsbS, Psb27, PetF and PetC) were downregulated in PO-type patchouli. These downregulated genes could decrease the light utilization efficiency in PO-type patchouli.

Chlorophyll (Chl) is the material basis of photosynthesis in plant leaves, and a reduction in the chlorophyll content will inhibit the capture and utilization of light energy by plants. The synthesis of chlorophyll directly affects photosynthetic efficiency [37]. In the pathway of porphyrin and chlorophyll metabolism, HemA (glutamyl-tRNA reductase), HemH, CAO (chlorophyllide an oxygenase), and ChlH (magnesium chelatase subunit H) genes play important roles in plant chlorophyll synthesis [38, 39]. Among them, HemA is the initial enzymatic step in the biosynthesis of chlorophyll [40, 41]. HemH is a key enzyme that regulates the pathway of heme synthesis, and the accumulation of heme affects the synthesis of chlorophyll [42, 43]. ChlH is a multifunctional protein with roles in plastid-to-nucleus and plant hormone signal transduction pathways [32]. As a subunit of Mg-chelatase, ChlH catalyses the conversion of protoporphyrin IX (Proto) to Mg-protoporphyrin IX (MgProto), a key regulatory step of chlorophyll biosynthesis [44]. The results of chlorophyll content analysis showed that the content of chl b in the PO-type was lower than that of the PA-type. CAO is responsible for Chl b synthesis [45]. Modifying the activity of CAO, an enzyme that participates in the synthesis of chl b, can alter the size and quantity of LHCII antennas since Chl b is their main constituent [46]. A study on tobacco demonstrated that CAO overexpression not only increased the Chl b content and the antenna size but also enhanced the efficiency of energy capture and its utilization at limiting as well as saturating light intensities when we measured PSI and PSII reactions separately [47]. The DEGs encoding HemA, HemH, ChlH, and CAO were downregulated in the PO-type, which would possibly influence the synthesis of chlorophyll and then lead to a decrease in leaf photosynthetic efficiency.

In addition to chlorophyll, carotenoids are essential components of all photosynthetic organisms. In plants, carotenoids can capture light energy and protect chlorophyll from photo-oxygen damage caused by intense light [48]. It is also a part of the chlorophyll-binding protein in the antenna system and reaction centre. The detection results of carotenoid content showed that the content of carotenoids in PO-type patchouli was even lower than that in PA-type patchouli. Phytoene synthase is a major rate-limiting enzyme in carotenoid biosynthesis, and its activity effectively determines the metabolic flux to carotenoids [49]. Lycopene β-cyclase (LCYB) is a key enzyme involved directly in the synthesis of α-carotene and β-carotene through the cyclization of lycopene [50]. Reduction of LCYB expression could decrease photosynthetic efficiency and plant biomass [51]. In the carotenoid synthesis pathway, 13 DEGs encoding phytoene synthase (crtB), 9-cis-epoxycarotenoid dioxygenase (NCED), capsanthin synthase (CCS1), beta-carotene hydroxylase (crtZ), zeaxanthin epoxidase (ZEP) and LCYB were downregulated in PO-type patchouli. Therefore, the differential gene expression levels in the carotenoid synthesis pathway could affect carotenoid content in two chemotypes of patchouli.

In summary, PA-type patchouli, compared to PO-type patchouli, could possibly enhance photosynthesis and increase yield by modulating genes involved in the pathway of photosynthesis, photosynthesis-antenna proteins, porphyrin and chlorophyll metabolism, and carotenoid biosynthesis.

## Conclusions

Physiological and biochemical indicators were measured individually on the two chemotypes of *P*. *cablin*, while UPLC-MS/MS was utilized for hormone metabolite detection. Transcriptomes and proteomes were integrated for conducting a combined analysis. The study revealed a significant difference in the content of photosynthetic pigments between the two chemotypes of *P*. *cablin*. Moreover, several pathways including photosynthesis, photosynthesis-antenna proteins, porphyrin and chlorophyll metabolism, and carotenoid biosynthesis showed differentially expressed genes. These findings suggest that the two chemotypes may have distinct photosynthetic efficiencies, leading to differences in plant morphology and yield. Ten phytohormone metabolites with significant differences were identified, mainly CKs such as zeatin, JAs, and GAs. The signaling function of JA was able to stimulate the synthesis of patchouli alcohol. The two chemotypes exhibited dissimilarities in plant morphology, leaf size, as well as patchouli alcohol content. The differences between the two chemotypes of *P*. *cablin* might be attributed to variations in hormone levels, photosynthetic efficiency and gene expression along the sesquiterpene synthesis pathway. These findings help to gain a better understanding of the mechanism underlying the formation of the two chemotypes of *P*. *cablin*.

## Supporting information

S1 Table Identification of phytohormone metabolites in two chemotypes of *P*. *cablin*. (DOCX)

S1 Fig Correlation analysis between mRNA and protein expression. (TIF)

S1 File Go enrichment analysis of DEGs and DEPs. (XLSX)

S2 File KEGG enrichment analysis of DEGs and DEPs. (XLSX)
