# Supplementary material for: Comparative physicochemical, hormonal, transcriptomic and proteomic analyses provide new insights into the formation mechanism of two chemotypes of Pogostemon cablin
Source: PLoS One. 2023 Sep 22;18(9):e0290402. doi: 10.1371/journal.pone.0290402 (PMC10516424; doi:10.1371/journal.pone.0290402)
Supplement: S1 Table — (DOCX) [file pone.0290402.s001.docx]

S1 Table identification of phytohormone metabolites in two chemotypes of *Pogostemon cablin*.

| Plant hormone | Compound name |  |  | class |
| --- | --- | --- | --- | --- |
| 2MeSiP | 2-methylthio-N6-isopentenyladenine riboside | | | CKs |
| DHZR | dihydrozeatin-9-riboside | |  | CKs |
| GA15 | Gibberellin A15 |  |  | GAs |
| GA9 | Gibberellin A9 |  |  | GAs |
| H2JA | dihydrojasmonic acid | |  | JAs |
| IP | N^6^-(Δ^2^-isopentenyl) adenine | |  | CKs |
| JA | jasmonic acid |  |  | JAs |
| OPC-4 | (Z)-4-[3-oxo-2-(pent-2-enyl) cyclopentyl] butanoic acid | | | JAs |
| 2CltZ | 2-chloro-trans- zeatin | |  | CKs |
| 2MeSiPR | 2-methylthio-N6-(Δ2-isopentenyl) adenosine | | | CKs |
| cZR | cis-zeatin-riboside |  |  | CKs |
| GA19 | Gibberellin A19 |  |  | GAs |
| GA3 | Gibberellin A3 |  |  | GAs |
| GA7 | Gibberellin A7 |  |  | GAs |
| JA-ILE | Jasmonyl-L-isoleucine | |  | JAs |
| MEJA | methyl jasmonate |  |  | JAs |
| mT9G | meta-topolin-9-glucoside | |  | CKs |
| pT9G | para-topolin-9-glucoside | |  | CKs |
| 2MeScZR | 2-methylthio-cis-zeatin riboside | |  | CKs |
| ABA | Abscisic acid |  |  | ABAs |
| ABA-GE | ABA-glucosyl ester |  |  | ABAs |
| cZ | cis-zeatin |  |  | CKs |
| cZ9G | cis-Zeatin-9-glucoside | |  | CKs |
| cZROG | cis-Zeatin-O-glucoside riboside | |  | CKs |
| DHZ7G | dihydrozeatin 9-riboside-N7-glucoside | |  | CKs |
| DHZROG | dihydrozeatin 9-riboside O-glucoside | |  | CKs |
| GA1 | Gibberellin A1 |  |  | GAs |
| iP7G | N6-isopentenyl-adenine-7-glucoside | |  | CKs |
| iP9G | N6-isopentenyl-adenine-9-glucoside | |  | CKs |
| IPR | N6-isopentenyladesine | |  | CKs |
| JA-Val | N-[(-)-Jasonoyl]-(L)-valine | |  | JAs |
| OPC-6 | (Z)-6-[3-oxo-2-(pent-2-enyl) cyclopentyl] hexanoic acid | | | JAs |
| OPDA | oxophytodienoic acid | |  | JAs |
| oT | ortho-topolin |  |  | CKs |
| tZOG | trans-zeatin-O-glucoside | |  | CKs |
