# Supplementary figures and images for: Comparative physicochemical, hormonal, transcriptomic and proteomic analyses provide new insights into the formation mechanism of two chemotypes of Pogostemon cablin
Source: PLoS One. 2023 Sep 22;18(9):e0290402. doi: 10.1371/journal.pone.0290402 (PMC10516424; doi:10.1371/journal.pone.0290402)

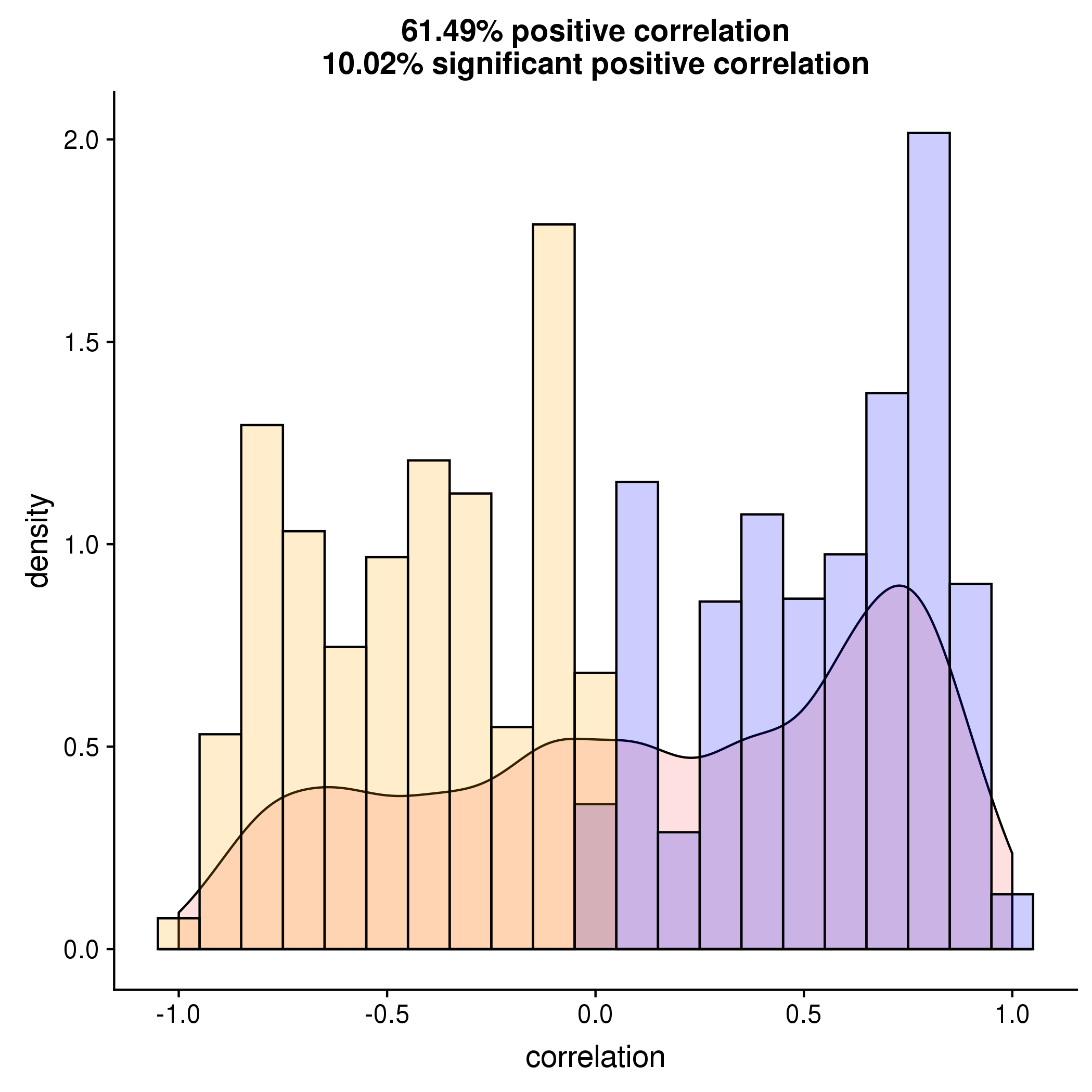

Supplement: S1 Fig — (TIF) [file pone.0290402.s002.tif]
